# COVID-19 Encephalopathy: Delayed Onset in Unvaccinated Patients

**DOI:** 10.7759/cureus.27932

**Published:** 2022-08-12

**Authors:** Dana Heller, Ramesh Pandit, Trupti Pandit, Gregory P Morris

**Affiliations:** 1 Medicine, Crozer-Chester Medical Center, Upland, USA; 2 Medicine, Independent Researcher, Philadelphia, USA; 3 Hospital Medicine, University of Pennsylvania/Chester County Hospital, Philadelphia, USA; 4 Pediatrics/Hospital Medicine, Nemours Children's Health and Inspira Medical Center, Glen Mills, USA; 5 Infectious Disease, Philadelphia College of Osteopathic Medicine (PCOM), Philadelphia, USA

**Keywords:** unvaccinated, duration of hospital stay, neurological complication, subacute, elderly population, altered mental status evaluation, covid-19 vaccination, covid-19-related encephalopathy

## Abstract

COVID-19 infections have a broad spectrum of severity, with more severe symptoms observed in elderly patients, patients with underlying comorbidities, and patients with unvaccinated status. This case series aims to highlight two cases of unvaccinated patients who developed COVID-19 encephalopathy, contrasted with a vaccinated patient with similar risk factors. This article highlights the unique characteristics of COVID-19 encephalopathy to guide clinicians while approaching the broad diagnosis of acute encephalopathy or altered mental state in hospitalized patients. Current literature was reviewed and summarized the information available regarding encephalopathy separate from the more complex encephalitis and encephalomyelitis.

## Introduction

COVID-19 encephalopathy is an altered brain function secondary to COVID-19 viral infection. It can present with various symptoms, but most commonly, altered consciousness, inability to communicate or understand language, confusion, slowness, and delirium, with a lack of imaging evidence of tissue damage. This is separate from encephalitis, acute disseminated encephalomyelitis, and acute necrotizing encephalopathy, although being part of the same spectrum of clinical presentation [1]. 

Pathogenesis

The pathogenesis of the COVID-19 infection and its invasion of the CNS is multifactorial. One autopsy case study demonstrated the presence of SARS-CoV-2 viral particles in the capillary endothelium and neurons in the frontal lobe [2]. Microscopy found endothelial cells that had endocytosed COVID-19 viral particles, which presents evidence of COVID-19's ability to cross the blood-brain barrier. Coupled with the severe inflammatory reaction accompanying the viral infection, it likely also increases the permeability of the blood-brain barrier [3]. The Trojan-horse mechanism is another theory, most commonly seen with HIV, where viruses have been shown to infect leukocytes and other WBCs that also express ACE2 protein [4]. It has been demonstrated that T-lymphocytes have allowed SARS-CoV-2 infection but not viral replication. These cells may also pass through a more permeable endothelial lining [3]. Profound hypoxia likely also plays a part in COVID-19's effect on mental status, causing delirium and encephalopathy. Infections causing cytokine storms and sepsis, which promote cytokines such as IL-6, 8, 10, and TNF alpha, have also been associated with hysterical states, most likely from the intense inflammatory reaction of the body [5]. Lastly, the metabolic derangements caused by COVID-19, such as hyponatremia, acute kidney injury, and elevated liver function tests, may individually affect mentation and delirium, resulting in metabolic encephalopathy [6]. 

Methodology

This study examines two cases of unvaccinated patients, from two Philadelphia area hospitals, who were admitted with COVID-19 and later developed acute altered mental status (encephalopathy) as well as new-onset atrial fibrillation (AFib) around similar timelines, comparing lab studies, disease progression, and hospital length of stay with the severity and duration of COVID-19 encephalopathy. The third case included provides a comparison between vaccinated and unvaccinated patients in regards to the disease severity as well as outcomes. Literature was reviewed with a search done using the PubMed dataset, with search terms "COVID" and "encephalopathy."

## Case presentation

Case #1

A 75-year-old male with a past medical history (PMH) of chronic kidney disease stage III with baseline creatinine of 2.4, diastolic heart failure, and deep vein thrombosis on Eliquis presented due to three days of generalized weakness and intermittent cough and was found to be COVID-19 positive. The patient denied shortness of breath, chest pain, abdominal pain, nausea, vomiting, or diarrhea. In the ED, vital signs on initial presentation were blood pressure: 128/88, heart rate: 66, respiratory rate: 22, oxygen saturation: 86% on room air, and 100% on a two-liter nasal cannula. In the ED, the patient was noted to have AFib with the rapid ventricular rate (RVR) at 146/min without change in O2 requirements or hypotension. No prior history of AFib was reported. He was started on a Cardizem infusion for rate control, with remdesivir and dexamethasone for COVID-19.

During the initial few days of hospitalization, he was verbal, awake, alert, and oriented (AAO) to person, place, time, and event with no deficit in executive function. By day three of admission, he reported feeling better regarding his weakness and malaise. He was also saturating SpO2 >94% on room air. The following night, the patient was more confused than normal. The patient was AAOx2, not oriented to time, and was not following commands. The next evening, he began refusing his medications. The following day (day six of hospitalization/day nine of COVID-19 symptoms), the patient appeared more altered, not responding to questions and became nonverbal, reacting only with grunts and appearing agitated. CT showed no intracranial hemorrhage, and EEG showed no active seizures. The patient underwent MRI due to his altered mental status, which showed four small foci of diffusion restriction in the right cerebral hemisphere; this finding was new compared to imaging one year prior. The neurologist opined that these MRI changes could not account for his acute change in mental status and global encephalopathy. He remained afebrile with normal blood pressure, and blood cultures were negative after five days. Thus, sepsis was effectively ruled out. Key lab results and imaging findings are detailed in Table 1.

**Table 1:** Case #1: Lab and imaging results on pertinent days. CMP: Complete metabolic panel; CO2: Carbon dioxide; AST: Aspartate transaminase; ALT: Alanine transaminase; PT/INR: Prothrombin time/International normalized ratio; ESR: Erythrocyte sedimentation rate; CRP: C-reactive protein; HS: High sensitivity; BNP: B-type natriuretic peptide; EEG: Electroencephalogram.

Lab results\day	Day of admission	Encephalopathy onset	Encephalopathy regression
WBC	9.5	10.9	10.6
Hemoglobin	13.2	12.3	7.6
Platelets	310	300	287
CMP			
Sodium	135	138	141
Potassium	3.6	3.6	3.9
CO2	24	24	22
Anion gap	11	9	8
Creatinine	2.3	2.8	2.2
Glucose	278	144	136
AST	14		
ALT	5		
Misc.			
PT/INR	15/1.2		
ESR	94	67	
CRP	106	76.7	43.9
D-Dimer	1.73	1.24	
Procalcitonin	0.54		0.44
HS-Troponin	N/A		
BNP	109		93
COVID-19 PCR Swab	Positive		
Urine analysis	Glucose +		
Lactic acid	1.7		
Blood culture		Negative	
EEG		Negative	
CT Head	No acute abnormalities		
Chest X-ray	Low lung volumes, no focal consolidation		
MRI Brain		Four small foci of diffusion restriction, new compared to prior.	

The patient continued to be nonverbal, grunting, requiring 1:1 monitoring for the next five days. The patient remained the same with slight improvement until the morning of hospital day 12, when he could respond to commands by lifting his arms or legs. The following day he was able to speak again and was AAOx3, returning mentation close to the baseline. The patient was eventually discharged in stable condition with a total length of stay of 18 days.

Case #2

A 78-year-old unvaccinated female with PMH of coronary artery disease, status post stent placement, iron deficiency anemia, hypertension, hyperlipidemia, smoking, depression, anxiety, and recent COVID-19 exposure three weeks ago, presented to the ED with shortness of breath. She had chills and a runny nose for two weeks but no fever with decreased oral intake for a few days before presentation. The patient appeared confused and could not properly respond to questioning, responding with "I do not remember" to all history questions. Per family members, the patient's shortness of breath started one day prior and was associated with a dry cough. The patient was reported to be at baseline mentation one day before the presentation.

The patient was admitted to the hospital for acute delirium secondary to COVID-19. Over the next few days, the patient continued to develop worsening mental status, becoming more agitated and confused, removing her oxygen and IV line. On day three of admission (day 16 of COVID-19 symptoms), the patient was AAOx0, grunting, and very agitated, requiring melatonin, Risperdal, and anxiolytic medication injections to control her agitation. The following evening, on day four of hospitalization (day 17 of COVID-19), the patient developed Atrial Fibrillation with RVR requiring multiple doses of labetalol and a Cardizem infusion. In addition, the patient experienced worsening O2 saturation and needed 10 Lt/min O2 on a non-rebreather mask. The following day, the patient was noted to have agitation, worsening acute encephalopathy, and worsening O2 requirement, with the patient requiring a 60 Lt high-flow nasal cannula. Incidentally, the patient was found to have a hemoglobin of 6.4, requiring transfusion with one unit of packed RBCs and an IV iron infusion. Hemoglobin remained stable during the rest of the inpatient stay. 

The patient continued to be AAOx0, nonverbal, and non-responsive to commands with constant grunting and agitation. The patient eventually required total parenteral nutrition (TPN) due to altered mentation and sustained inability to take oral feeds. On day nine of hospitalization (COVID-19 day 22), the patient appeared to respond to verbal commands with a more alert appearance. On day 10 of hospitalization (day 23 of COVID-19), the patient was AAOX3-4, able to respond to commands, answer questions, and communicate with the team. The patient was effectively weaned off oxygen over the next few days and started physical therapy to regain strength and mobility. She was discharged home with no O2 requirements and with home physical therapy on hospital day 16 (day 29 of COVID-19). Relevant lab results during the hospitalization are listed in Table 2.

**Table 2:** Case #2: Lab and imaging results on pertinent days. CMP: Complete metabolic panel; CO2: Carbon dioxide; AST: Aspartate transaminase; ALT: Alanine transaminase; PT/INR: Prothrombin time/International normalized ratio; ESR: Erythrocyte sedimentation rate; CRP: C-reactive protein; HS: High sensitivity; BNP: B-type natriuretic peptide; UA: Urinalysis.

Lab Test	At Admission	Encephalopathy Onset	Encephalopathy Regression
WBC	13.3	20.6	13
Hemoglobin	8.5	7.6	8.1
Platelets	437	594	392
CMP		22	25
Sodium	135	146	145
CO2	20	22	25
Anion Gap	13	14	4
Creatinine	1.4	0.9	0.7
Glucose	106	117	150
AST	35		
ALT	13		
PT/INR	14.5/1.2		
CRP	140.2	47.7	<5.0
D-Dimer	1.67	1.77	
Procalcitonin	0.55	0.15	0.05
HS Troponin	15		
BNP	177		
COVID-19 PCR Swab	Positive		
UA	Unremarkable		
Lactic Acid	0.9	2.9	1.9
Blood Culture	Negative		
Sputum Culture		Negative	
CT Head	No acute abnormalities		
Chest X-ray	Diffuse opacities throughout lung fields, prominent in the hilar region		

Case #3

A 94-year-old female with PMH of dementia, hypothyroid, hypertension, CKD, and AFib five years ago presented to the ED with a three-day history of repeated vomiting and decreased oral intake. The primary caretaker provided further details of the patient's history and presentation at home. The patient was noted to have lethargy and reduced appetite for three days but remained at her baseline level of alertness, oriented to place and person. The patient had sick contact at home and had a positive COVID -19 antigen test at home the day before the presentation. The patient had received two doses of the Moderna vaccine, with the last dose about four months prior, and was not due for the booster dose.

On presentation, the patient was found to have AFib with RVR at a heart rate of 150; and received a Cardizem bolus and started on the Cardizem infusion. She transiently desaturated to 90%. The patient was admitted to the hospital for AFib with RVR and acute hypoxic respiratory failure, requiring 2-3 liter oxygen via nasal cannula, secondary to COVID-19 pneumonia. Key lab results and imaging findings are detailed in Table 3 below. The patient was started on remdesivir and dexamethasone, and throughout the hospital stay, the patient's mental status remained at her baseline with dementia, being oriented to place and person. The patient has transitioned to metoprolol 100 mg oral daily dosing. Incidentally, the patient was noted to have GI bleed, with a fecal occult blood test (FOBT) positive with hemoglobin drop from 11 to 8, that was managed conservatively with proton pump inhibitors, and plan for outpatient follow up with gastroenterologist as hemoglobin remained stable for rest of the hospital stay. The patient completed her remdesivir course and was discharged home on day 5. 

**Table 3:** Case #3: Lab and imaging results. CMP: Complete metabolic panel; CO2: Carbon dioxide; AST: Aspartate transaminase; ALT: Alanine transaminase; PT/INR: Prothrombin time/International normalized ratio; ESR: Erythrocyte sedimentation rate; CRP: C-reactive protein; HS: High sensitivity; BNP: B-type natriuretic peptide; UA: Urinalysis.

Lab tests	
CBC	Admission
WBC	8.3
Hemoglobin	11.1
Platelets	303
CMP	
Sodium	136
CO2	24
Anion Gap	11
Creatinine	1.5
Glucose	80
AST	18
ALT	11
PT/INR	N/A
CRP	41.4
D-Dimer	1.67
Procalcitonin	0.17
HS-Troponin	80
BNP	N/A
COVID-19 PCR Swab	Positive
Urine Analysis	Negative
Lactic Acid	N/A
Blood Culture	N/A
Sputum Culture	N/A
CT Head	N/A
Chest X-ray	Bilateral lower lobe multifocal infiltrates

## Discussion

The literature surrounding COVID-19 encephalopathy symptom's presentation and onset is somewhat limited, most likely due to its vague presentation and extensive comorbidities these patients usually have. Some cases have shown neurological manifestations of COVID-19 symptoms without respiratory symptoms. As in case #2, the patient reported increasing confusion after two weeks of viral prodrome symptoms. Most patients did not require invasive or noninvasive ventilation, except for needing a high-flow nasal cannula. It is important to note that some severe forms of COVID-19 may only be evident through neurologic sequelae without the expected respiratory failure. Siddiqui AF et al. reported a middle-aged woman presenting with a new onset tonic-clonic seizure, who was later diagnosed with COVID-19 encephalitis without any significant respiratory symptoms [7]. COVID-19 encephalitis presentation does vary far beyond encephalopathy and possible seizure. All three cases we reported had returned to baseline mental function, but a detailed cognitive evaluation was not performed, limiting the long-term evaluation. The key findings of our patients are summarized in Table 4.

**Table 4:** Comparing key features among cases. CKD: Chronic kidney disease; CHF: Congestive heart failure; DVT: Deep venous thrombosis; CAD: Coronary artery disease; HTN: Hypertension; HLD: Hyperlipidemia; AFib: Atrial fibrillation; LOS: Length of stay.

Patient	Age/Sex	Vaccination Status	Comorbidities	Complications	Onset of Neurological Symptoms (days)	Neurological Symptoms	Hospital LOS (days)
Case #1	75 years, male	Unvaccinated	CKD, CHF, DVT	AFib	6	Delirium, agitation	18
Case #2	78 years, female	Unvaccinated	CAD, HTN, HLD, depression	AFib	13	Delirium, agitation	16
Case #3	94 years, female	Vaccinated	Dementia, HTN, CKD, AFib	AFib	N/A	No acute change	5

As mentioned previously, there are a few different mechanisms described in which COVID-19 may cross the blood-brain barrier and result in neurological symptoms. Mao L et al. found that more than 1/3rd of the patients with COVID-19 had neurological manifestations, with 7.5% reporting altered consciousness [8]. Cerebral frontal hypometabolism and cerebellar hypermetabolism were noted as per positron emission tomography (PET) assessment by Delorme C et al., with an immune-mediated mechanism suspected based on the improvement reported in symptoms with immunotherapy [9].

Delorme C et al. also reported a delay of 0-12 days between COVID-19 symptoms and the onset of neurological symptoms [9]. Similar findings were noted in individual case reports of patients presenting with acute encephalopathy with unremarkable CT head within 1-2 days of COVID-19 symptoms, identical to our third case [10]. As per the systematic review by Garg RK et al. [11], encephalopathy was commonly noted in critically ill patients. A total of 69% of patients reported having delirium, with the onset of encephalopathy between 5 and 14 days of illness. Most patients in that review required ICU stay. Encephalitis cases were included in the study, limiting the direct comparison with cases in our report. The critically ill patients often have non-COVID-19-related factors resulting in encephalopathy [12]. Mild-to-moderate forms of encephalopathy often go unreported or are considered metabolic [13]. 

Chronic encephalopathy or long-term neurological impact has been reported in patients with COVID-19 [14-17]. As noted by Vialatte de Pémille C et al., acute and subacute cognitive dysfunction have been reported, similar to our patients [14]. However, they reported significant improvement in cognitive test scores at three months. One-third of patients with severe COVID-19 reported neurological disturbances, but similar findings were noted with ICU admission or ventilation dependence without COVID-19 [15, 16]. One such retrospective study reported an incidence of dementia at 0.67% six months after COVID-19 infection, while those requiring ICU admission with 1·74% incidence [17]. Vaccination against COVID-19 has been reported to protect against SARS-CoV2 infection, especially protecting against severe symptoms, including encephalitis and long COVID-19 symptoms [18, 19]. We estimate that the protection also helps reduce the occurrence and severity of encephalopathy. Additional studies comparing cognitive function before and after COVID-19 encephalopathy will be needed to assess the long-term impact.

## Conclusions

Elderly patients with comorbidities and unvaccinated COVID-19 status are more vulnerable to developing COVID-19 encephalopathy, even with mild-to-moderate respiratory disease. Vaccination against COVID-19 is likely to prevent the onset of severe or prolonged encephalopathy, as evidenced in case 3. Again, this reinforces the need for vaccination to prevent COVID-19 disease and related comorbidities. Especially in populations at risk, initial and booster vaccination is essential. Although the resolution of most neurological symptoms is promising, long-term follow-ups are advised for patients with encephalopathy during hospitalization. The COVID-19 infection is associated with an increased incidence of neurological disorders in the six months of follow-up. The health care providers should educate the patients and families regarding the subacute and long-term risks associated with COVID-19 at discharge.
